# A randomised controlled trial of structured nurse-led outpatient clinic follow-up for dyspeptic patients after direct access gastroscopy

**DOI:** 10.1186/1471-230X-9-12

**Published:** 2009-02-06

**Authors:** David Chan, Scott Harris, Paul Roderick, David Brown, Praful Patel

**Affiliations:** 1 Department of Gastroenterology, Southampton University Hospitals Trust, Southampton, UK; 2 Public Health Sciences and Medical Statistics faculty, Southampton University, Southampton, UK; 3 Pharmacology and Bio Medical Sciences faculty, Portsmouth University, Portsmouth, UK

## Abstract

**Background:**

Dyspepsia is a common disorder in the community, with many patients referred for diagnostic gastroscopy by their General Practitioner (GP). The National Institute of Clinical Excellence (NICE) recommends follow-up after investigation for cost effective management, including lifestyle advice and drug use. An alternative strategy may be the use of a gastro-intestinal nurse practitioner (GNP) instead of the GP. The objective of this study is to compare the effectiveness and costs of systematic GNP led follow-up to usual care by GPs in dyspeptic patients following gastroscopy.

**Results:**

Direct access adult dyspeptic patients referred for gastroscopy; without serious pathology, were followed-up in a structured nurse-led outpatient clinic. Outcome measurement used to compare the two study cohorts (GNP versus GP) included Glasgow dyspepsia severity (Gladys) score, Health Status Short Form 12 (SF12), ulcer healing drug (UHD) use and costs. One hundred and seventy five patients were eligible after gastroscopy, 89 were randomised to GNP follow-up and 86 to GP follow-up. Follow-up at 6 months was 81/89 (91%) in the GNP arm and 79/86 (92%) in the GP arm. On an intention to treat analysis, adjusted mean differences (95%CI) at follow-up between Nurse and GP follow-up were: Gladys score 2.30 (1.4–3.2) p < 0.001, SF12 140.6 (96.5–184.8) p =< 0.001 and UHD costs £39.60 (£24.20–£55.10) p =< 0.001, all in favour of nurse follow-up.

**Conclusion:**

A standardised and structured follow-up by one gastrointestinal nurse practitioner was effective and may save drug costs in patients after gastroscopy. These findings need replication in other centres.

## Background

Dyspepsia is a common complaint that leads to significant health care costs [1-3]. The management of dyspepsia and its related causes has progressed in recent years. In England, the National Institute of Clinical Excellence (NICE), published recommendations (2004) to promote cost effective management [4]. Key recommendations were for follow-up, after direct access gastroscopy to maintain minimum effective therapy, to provide lifestyle advice and to perform an annual review. A large proportion of these patients are managed within primary care but the effectiveness of such care is unknown [5]. A contributory factor may be the limited time of a general practitioner's (GP) consultation and prioritisation of GP workload to more serious conditions. Other health-care professionals, such as gastrointestinal nurse practitioners (GNP), may be capable of taking on this role and provide more appropriate care within available resources.

This study describes a randomised controlled trial, which compared the effectiveness and impact on acid suppressant use and costs of a systematic GNP-led follow-up in an outpatient clinic to usual care by GPs, in patients with dyspepsia after direct access gastroscopy.

## Methods

All GP surgeries in the catchment area of a teaching hospital referral centre (Southampton University Hospital Trust) were included. All direct access referrals for gastroscopy were screened to exclude those with sinister symptoms i.e. dysphagia, vomiting, anaemia, rapid weight loss or those with history of gastric surgery. Patients were consented at the point of recruitment. Trained medical endoscopists performed the gastroscopy procedure. Patients found to have peptic ulcer, tumour, severe oesophagitis (grade C and D), Barrett's oesophagus and anatomical abnormality were excluded. Patients included were those with mild gastro-oesophageal reflux disease (GORD – non-erosive or grade A and B oesophagitis, hiatus hernia), non-ulcer dyspepsia (NUD) (mild and moderate gastritis or duodenitis) and those with normal findings.

Baseline details of socio-demographic factors, education, self-reported height and weight, smoking, alcohol (current versus non-drinker) and ulcer healing drugs (UHD) used in the past 6 months were collected by interview of all patients presenting for elective gastroscopy at Southampton University Hospitals Trust for the period between May 2002 to May 2004. All patients also completed two validated questionnaires relating to the past 6 months: the Glasgow Dyspepsia severity scores (Gladys) and the Health Status Short Form 12 (SF-12) [6,7]. After gastroscopy, endoscopists maintained their routine practice in giving verbal and written advice to patients and documented treatment recommendation to GPs in a formal report. Patients eligible for entry after endoscopy were randomised into intervention (GNP) and control (GP) groups, with a password protected, computer generated random number table. The endoscopists telephoned a separate office to obtain the follow-up status. The 'GNP' group was given one out-patient appointment. The 'GP' cohort was discharged and advised to see their GP.

In the nurse-led clinic, a full medical history was taken. The clinical management was structured, based on national and local guidelines, with reference to each patient's predominant symptoms. Patients were given counselling and lifestyle advice, supplemented with relevant locally devised leaflets i.e. reflux, non-ulcer dyspepsia, weight control, and an individualised treatment plan agreed with them. Further investigation such as the urea breath test, motility studies and barium meal were initiated if required, as per routine clinical practice. To ensure practice consistency and reproducibility, 'history taking' and 'lifestyle advice' proformas were devised and used.

### Follow-up

A researcher blinded to the patients' study status and diagnosis, interviewed all participants by telephone, at a pre-arranged time suitable to the patient, six months after randomisation. Data collected were Gladys dyspepsia score, SF-12 score, self-reported UHD used and weight and a patient satisfaction questionnaire.

### Drug use and cost

The use of UHDs for the six months before (baseline) and for 6 months after trial entry were summed and averaged according to class: Proton pump inhibitors (PPI) and Histamine_2 _receptor antagonists (H_2_RA) and strength (half and full-dose PPI). Half-dose PPI and H_2_RAs were grouped, as they were equivalent in costs. Drug use by month, based on quantity of tablets, was therefore grouped: 'full-dose PPI' vs 'half-dose PPI and H_2_RA' vs 'no treatment'. Twice daily full-dose PPI was counted as 2 months and alternate days use counted as half a month. On demand therapy was recorded according to number of tablet/s taken per week and multiplied by 4 to give a monthly quantity. UHD prices were taken for each specific UHD from Monthly Index of Medical Speciality (MIMS 2006) and Drug Tariff (2006).

### Statistical analysis

A sample size calculation indicated that a minimum of 186 patients, 93 in each group, would be needed to detect a 1 point of improvement on the Gladys dyspepsia scale with 80% power at the 5% significance level. This calculation was based on a standard deviation of 2.85 that came from a pilot study of 30 cases presenting for gastroscopy in which the Gladys scores were obtained before the procedure.

The two groups were compared by the change from baseline to month 6 in the key outcome variables – Gladys score, SF12 and overall UHDs cost, adjusted for baseline values by including the baseline levels of the outcome in the ANOVA as a covariate; p < 0.05 was taken as being significant. Intention to treat analysis was undertaken by assuming the 15 patients with no 6 month follow-up data did not change from baseline. The component scales of SF12 and Gladys were analysed with p values calculated using the Mann-Whitney or Chi-square test where appropriate; a p value of < 0.01 was taken because of multiple testing.

Local ethics committee approval was obtained (reference no. 050-02) and the study registered with the Southampton University Hospitals NHS Trust Research Department.

## Results

Over a 2 year period, on the elective direct access gastroscopy lists at which the GNP could attend, 199 unselected patients were approached and 196 (98.5%) were recruited. One hundred and seventy five (89.3%) patients were eligible after investigation. Of the 21 ineligible patients, 16 did not meet the criteria (Barrett's oesophagus: 6, oesophagitis grade C: 2, oesophageal stricture: 1, peptic ulcer disease: 3, possible cancer: 1). Three cases were deemed unsuitable by the endoscopist, as symptoms were attributed to other conditions (rhinitis 1, angina 2). Two did not have the procedure (failed intubation 1, food in stomach 1).

The 175 patients were randomised, 86 to GP follow-up (GP) and 89 to nurse follow-up (GNP). There were no obvious differences in the baseline characteristics of the two groups in age, BMI, smoking, alcohol and education status (see Table 1). The baseline Gladys scores (high scores equal higher burden of disease and symptoms) were similar (10.0 vs 9.9) but the SF12 scores (672.0 vs 627.7) were higher (high scores equal better health) in the GP group (see Table 1). The cost of UHD used, 6 months prior to the investigation, was lower in the GP group (£52.4 vs £59.5).

**Table 1:** Baseline characteristics of patients randomised to 'GP' and 'GNP' groups.

		Treatment group	
		
		GP N = 86	GNP N = 89
Age yrs	Mean (SD)	48.4(12.8))	50.2 (13.9)

Gender	Male	42 (49)	44 (49)

BMI kg/m^2^	Mean (SD)	27.5 (5.0)	27.0 (5.8)

Smoking status	Current	23 (27)	24 (27)
	Ex	43 (50)	42 (47)
	Non	19 (22)	23 (26)
	Unknown	1 (1)	0 (0)

Alcohol	Drinker	76(88)	78 (88)

Education (grouping)			
No qualifications		30 (35)	35 (39)
'O' levels & vocational qualifications		22 (26)	20 (22)
'A' levels to diplomas		11 (13)	12 (13)
Professional, 1^st ^& higher degrees		23 (27)	22 (25)

Gladys score	Mean (SD)	10.0 (3.0)	9.9 (3.2)

SF12	Mean (SD)	672.0 (157.3)	627.7 (197.930)

UHD costs (based on following*)	Mean (SD) in £	52.4(47.6)	59.5(46.3)

*UHD types in previous 6 months	Months of use		
Full dose PPI		1.4(2.0)	1.5(2.0)
Half dose PPI or H2RA		1.1(1.9)	1.5(2.2)
None		3.4(2.2)	3.0(2.2)

* Details of grouping in 'Statistical analysis'.

Figures are number (%) unless specified.

### Intervention

**Figure 1:**
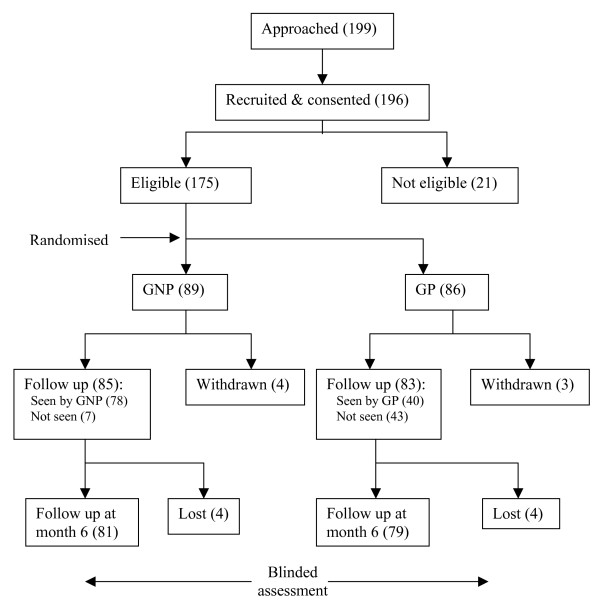
**This figure shows the number of patients approached, recruited, randomised to follow-up and seen by the Gastrointestinal nurse practitioner and GPs in both cohorts**.

Early withdrawals (GP n = 3 GNP n = 4) after randomisation were experienced in both groups (Figure 1), Three in the 'GP' group decided not to see their GP. The four in the 'GNP' group were due to work commitments (2), leaving the area (1) and after own GP consultation (1). 78/89 (88%) actually attended the GNP clinic compared to only 40/86 (47%) in the GP arm.

In the GP arm, 12 (30.0%) recalled not having had their gastroscopy findings explained, 23 (57.5%) were given lifestyle advice and 5 (12.5%) received dyspepsia information leaflets by their GPs. Most patients found the information given to them of limited use and most of them rated it ineffective in changing their lifestyle. In contrast, all those who attended the nurse-led clinic said that their gastroscopy findings were explained and receiving lifestyle advice with leaflets. Furthermore, most patients reported that this information enabled them to make positive lifestyle changes.

### 6 month follow-up

The response rate was high: 79/86 (92%) in the GP arm and 81/89 (91%) patients in the GNP arm (see Figure 1). Both groups had symptom improvement at 6 month follow-up but this was significantly better in the 'GNP' group (see Table 2) showing the Gladys score difference adjusted for baseline was 2.3 (1.4–3.1, p < 0.001). The SF12 data also showed that the nurse group had the greatest improvement (140.7 (96.5–184.8) p < 0.001). 'GP' drug costs (per patient per week) rose by £19.30 and 'GNP' group fell by £24.30 over the study period with a significant difference at 6 months (£39.6 (£24.2–£55.1) p < 0.001).

**Table 2:** Comparison of key outcomes at 6 month follow-up between the 'GP' and 'GNP' groups.

			Unadjusted		Adjusted*	
			
	GP (n = 86)	Nurse (n = 89)	GP -- GNP		GP -- GNP	
	
	Mean score (SD)		Mean difference (95% CI)	p-value	Mean difference (95% CI)	p-value
**SF 12**	634.8 (195.8)	754.5 (138.6)	-119.7 (-170.6, -68.9)	< 0.001	-140.7 (-184.8, -96.5)	< 0.001

**Gladys score**	7.2 (3.1)	4.9 (2.9)	2.3 (1.4, 3.2)	< 0.001	2.3 (1.4, 3.1)	< 0.001

**UHD costs (£)**	71.7 (63.1)	35.5 (48.8)	36.2 (19.3, 53.1)	< 0.001	39.6 (24.2, 55.1)	< 0.001

* Adjusted for baseline level using ANOVA.

Seven patients in each arm had a positive *H. pylori *test and received eradication therapy. The same number of patients (3) in each group had further tests. In the 'GP' group, 2 patients recalled having a urea breath test to confirm eradication success and 1 had abdominal ultra sound for epigastric pain; all had negative findings. Three patients in the 'nurse' group had 24-hour pH manometery studies, for volume reflux and severe GORD was confirmed. Two patients opted for surgical intervention. Criteria for further investigation by the nurse were according to routine clinical protocol for patients with severe and/or treatment resistant dyspeptic symptoms.

Of the nine domains in the Gladys questionnaire (see Table 3), four showed improvement in the 'GNP' group: reduced episodes of dyspepsia (p = 0.003), less dyspepsia interfering with normal activities (p < 0.001), lower doctor visits (though this would be anticipated due to the design), and less prescribed UHDs (p = 0.001); no significant differences were seen in the remaining five. Seven out of twelve domains in SF12 (see Table 4) showed significant improvement in the 'GNP' group.

**Table 3:** Comparison of individual Gladys questionnaire responses at 6 month follow-up.

		Treatment group		p value
			
		GP	GNP	
Health limited social activities	All of the time	0 (0)	0 (0)	0.225^†^
	Most of the time	1 (1)	2 (3)	
	A good bit of the time	1 (1)	0 (0)	
	Some of the time	5 (6)	2 (3)	
	A little of the time	6 (8)	4 (5)	
	None of the time	66 (84)	73 (90)	

Over the last 6 months how frequent were indigestion symptoms	Never	7 (9)	10 (12)	0.003^†^
	1 or 2 days	9 (11)	21 (26)	
	1 day per month	15 (19)	16 (20)	
	1 day per week	22 (28)	22 (27)	
	50% of days	12 (15)	7 (9)	
	Most days	14 (18)	5 (6)	

Did indigestion interfere with normal activities	Never	21 (27)	44 (54)	<0.001^†^
	Sometimes	45 (57)	34 (42)	
	Regularly	13 (17)	3 (4)	

Days off work due to indigestion in 6 months	None	74 (94)	79 (98)	0.236^†^
	1–7 days	3 (4)	1 (1)	
	More than 7 days	2 (3)	1 (1)	

Frequency of attending a doctor in 6 months	None	63 (80)	77 (95)	0.003^†^
	Once	6 (8)	2 (3)	
	Twice or more	10 (13)	2 (3)	
				
GP home visits due to indigestion in 6 months	None	79 (100)	81 (100)	1.000^†^
	Once	0 (0)	0 (0)	
	Twice or more	0 (0)	0 (0)	

Number of tests for indigestion in 6 months	None	76 (96)	78 (96)	0.975^†^
	Once	3 (4)	3 (4)	
	Twice or more	0 (0)	0 (0)	

Use of self-obtained medication in 6 months	Never	38 (48)	47 (58)	0.047^†^
	Less than 1/week	19 (24)	26 (32)	
	More than 1/week	22 (28)	8 (10)	

Use of prescribed drugs in 6 months	Never	15 (19)	31 (38)	0.001^†^
	1 month or less	8 (10)	14 (17)	
	1 to 3 months	8 (10)	8 (10)	
	More than 3 months	48 (61)	28 (35)	

^† ^P value from Mann-Whitney test.

Figures are number (%).

**Table 4:** Comparison of individual SF12 questions at 6 months follow-up.

		Treatment group		p value
			
		GP	GNP	
General health	Excellent	1 (1)	7 (9)	< 0.001^†^
	Very good	8 (10)	32 (40)	
	Good	33 (42)	26 (32)	
	Fair	35 (44)	15 (19)	
	Poor	2 (3)	1 (1)	

Health limited – Moderate activities	Yes, limited a lot	3 (4)	1 (1)	0.261^†^
	Yes, limited a little	15 (19)	12 (15)	
	No, not limited at all	61 (77)	68 (84)	

Health limited – Climbing several flights of stairs	Yes, limited a lot	6 (8)	2 (3)	0.021^†^
	Yes, limited a little	21 (27)	13 (16)	
	No, not limited at all	52 (66)	66 (82)	

Accomplished less than you would like in 4 weeks	Yes	27 (34)	8 (10)	< 0.001^‡^
	No	52 (66)	73 (90)	

Were limited in the kind of work or other activities in 4 weeks	Yes	22 (28)	7 (9)	0.002^‡^
	No	57 (72)	74 (91)	

Accomplished less than you would like	Yes No	22 (28) 57 (72)	6 (7) 75 (93)	0.001^‡^

Didn't do work or other activities as carefully as usual	Yes	15 (19)	4 (5)	0.006^‡^
	No	64 (81)	77 (95)	
				
Pain interfering with normal work in 4 weeks	Not at all	31 (39)	42 (52)	0.023^†^
	A little bit	24 (30)	28 (35)	
	Moderately	8 (10)	3 (4)	
	Quite a bit	11 (14)	8 (10)	
	Extremely	5 (60)	0 (0)	

Felt calm and peaceful	All of the time	5 (6)	5 (6)	0.024^†^
	Most of the time	17 (22)	31 (38)	
	A good bit of the time	7 (9)	4 (5)	
	Some of the time	35 (44)	36 (44)	
	A little of the time	11 (14)	2 (3)	
	None of the time	4 (5)	3 (4)	

Had a lot of energy	All of the time	1 (1)	3 (4)	0.002^†^
	Most of the time	13 (17)	24 (30)	
	A good bit of the time	7 (9)	12 (15)	
	Some of the time	33 (42)	29 (36)	
	A little of the time	14 (18)	10 (12)	
	None of the time	11 (14)	3 (4)	

Felt downhearted and low	All of the time	1 (1)	0 (0)	0.001^†^
	Most of the time	6 (8)	1 (1)	
	A good bit of the time	6 (8)	2 (3)	
	Some of the time	30 (38)	24 (30)	
	A little of the time	18 (23)	20 (25)	
	None of the time	18 (23)	34 (42)	

^† ^P value from Mann-Whitney test.

^‡^P value from Chi-square test.

Figures are number (%).

### Patterns of drug use

The baseline UHD use was well matched (see Table 1) between the 2 groups. Over the 6 month follow-up period the GNP group consumed less months of 'full-dose PPI' medication 1.5(0.8–2.1) p < 0.0001 and more had 'no treatment' 1.7(0.9–2.4) p < 0.001. There were similar months of 'half-dose PPIs and H_2_RAs'used in both groups (see Table 5).

**Table 5:** Comparison of monthly UHD use at 6 month follow-up between 'GP' and 'GNP' groups.

			Unadjusted		Adjusted*	
			
	GP (n = 86)	GNP (n = 89)	GP -- GNP		GP -- GNP	
	
Drug pattern in months	Mean score (SD)		Mean difference (95% CI)	p-value	Mean difference **(95% CI)**	p-value
**Full dose PPI **	2.0 (2.7)	0.6 (1.7)	1.4 (0.8, 2.1)	< 0.001	1.5 (0.8, 2.1)	< 0.001

**Half dose PPIs**	1.5 (2.4)	1.4 (2.3)	0.0 (-0.7, 0.7)	0.942	0.1 (-0.5, 0.8)	0.702

**No treatment**	2.5 (2.7)	4.0 (2.5)	-1.5 (-2.2, -0.7)	< 0.001	-1.7 (-2.4, -0.9)	< 0.001

* Adjusted for baseline level.

^† ^Including all H_2_RAs.

## Discussion

This is the first randomised controlled trial to investigate the effectiveness of nurse-led dyspepsia management compared to usual GP follow-up. A robust reproducible intervention to control symptoms and minimise medication was achieved by providing patients with appropriate information after OGD. The main findings were a significant reduction in symptom severity, an improvement in health related quality of life, and reduced anti-ulcer drug use and costs in the nurse-led group.

This trial followed established design principles with concealed allocation, minimal loss to follow-up, blinding of assessment and use of validated assessment instruments. It was a pragmatic study, which compared different professionals in different locations comparing current practice. The main limitation was the use of a single centre with only six-month follow-up. Longer follow-up would allow assessment of sustainability of the intervention, and increasing the number of centres would enhance generalisibility. A potential limitation was the lack of distinction between different diagnostic groups; with mixing of non-ulcer type and reflux-type symptoms though appropriate lifestyle advice was provided based on predominant symptoms. We did not undertake full economic costing but rather focused on drug costs as the major component. The overall cost-effectiveness of this strategy need further evaluation taking into account all cost elements i.e. clinicians' time, additional investigation, surgical intervention and administrative support.

Previous studies have shown the beneficial effect of gastroscopy on patients' symptoms [8-10]. The outcome of this study shows that the additional interventions in the nurse-led clinic had added benefit; by reducing UHD costs on the background of improved symptoms and well being. The major differences between this and other upper GI nurse-led studies include the use of valid end-points, randomised design and estimation of the impact on drug cost [11-13]. This study demonstrates that investigation is not the end point of dyspepsia management; rather it provides clinicians with the means to formulate a personalised treatment plan and the importance of explanation, counselling and patient empowerment in managing their illness.

A contributory factor to the greater effectiveness of nurse-led care might have been the low attendance rate (47%) of patients to see their GPs after endoscopy, compared to the almost universal attendance at the nurse clinic. This emphasizes the need for appropriate advice even if patients are found to have minor or normal findings. Patient perspectives on the nurse clinic were very positive. The structured approach supported by locally devised lifestyle leaflets to reinforce this advice, may have motivated patients to self-care more effectively e.g. avoid late meals, reduce portion size, smoking, alcohol and caffeine and may have contributed to the outcome differences [14-18].

The cost of drugs used had increased by approximately one third at six months review in the 'GP' group. This increase was consistent with the Gladys questionnaire in 'over-the-counter' drug use. Dyspepsia is a symptoms-complex disorder and although UHDs may be effective in controlling acid-related symptoms, their use for non-acid related disorders are inadequate [19-22]. Thus with advice on healthy lifestyle and stress management, non-acid related symptoms could be controlled more effectively, leading to UHD reduction. In a wider context, nurse-led follow-up could augment medical care, improve general health and release doctors' time to focus on more complex cases. This may be an important strategy as more diagnostic endoscopy is carried out in primary care, though the effectiveness of nurse-led care within the primary care setting would need to be tested. The study did not examine GPs' satisfaction with nurse-managed care, as it has been established in other specialist nurse-led services [23-25].

It is conceivable that the strategy could be transferable to other specialist areas with less complex, high volume illnesses, e.g. irritable bowel syndrome, where time and counselling skills are required in addition to support from both medical and nursing professional bodies [26-29]. Furthermore, this is an agenda in the NHS reform of the care of patients with chronic illness in the community [30].

## Conclusion

In conclusion, follow-up management of dyspepsia after direct access gastroscopy is variable. It can be standardised involving an experienced GNP, with sufficient time to give practical advice, which can empower patient to self-care effectively. This can lead to substantial health gains and reductions in drug costs in the community. This approach needs to be tested in other centres.

## Competing interests

The authors declare that they have no competing interests.

## Authors' contributions

DC conceived the study, participated in its design, acquisition of data, co-ordination and writing it up. SH participated in the design and performed the statistical analysis. PR participated in the design, coordination and critical revision of the study. DB participated in the design and critical revision of the study. PP conceived the study, participated in the design, co-ordination and critical revision of the study. All authors read and approved the final manuscript.

## Pre-publication history

The pre-publication history for this paper can be accessed here:

http://www.biomedcentral.com/1471-230X/9/12/prepub
